# Non-vitamin K Antagonist Oral Anticoagulants vs. Warfarin at Risk of Fractures: A Systematic Review and Meta-Analysis of Randomized Controlled Trials

**DOI:** 10.3389/fphar.2018.00348

**Published:** 2018-04-10

**Authors:** Zhi-Chun Gu, Ling-Yun Zhou, Long Shen, Chi Zhang, Jun Pu, Hou-Wen Lin, Xiao-Yan Liu

**Affiliations:** ^1^Department of Pharmacy, Renji Hospital, School of Medicine, Shanghai Jiaotong University, Shanghai, China; ^2^Department of Pharmacy, The Third Xiangya Hospital, Central South University, Changsha, China; ^3^Department of Cardiology, Renji Hospital, School of Medicine, Shanghai Jiaotong University, Shanghai, China

**Keywords:** non-vitamin K antagonist oral anticoagulants, warfarin, fractures, bone, meta-analysis, risk factors

## Abstract

Warfarin is a traditional oral anticoagulant for preventing thrombotic events in patients with atrial fibrillation (AF) and venous thromboembolism. Along with the widespread clinical use, the potential association between warfarin use and fracture risk have been addressed gradually. Non-vitamin K antagonist oral anticoagulants (NOACs), targeting thrombin or Xa factor, have been recommended as an optimal alternative due to their favorable property of thromboembolism prophylaxis and reduced bleeding risk. However, evidence of the fracture risk with NOACs use is limited. Therefore, the present study investigated this issue by a meta-analysis. Medline, Embase, Cochrane Library and the ClinicalTrials.gov Website were searched for randomized controlled trials (RCTs) that reported the fracture data of NOACs and warfarin. The primacy outcome was a composite of any fracture. Summary relative risks (RRs) and 95% confidence intervals (CIs) were calculated using random- or fixed-effects models according to between-study heterogeneity. Heterogeneity was assessed through *I*^2^ test and Q statistic, and the number of patients needed to treat (NNT) was calculated based on fracture incidence. Subgroup analyses were conducted according to individual NOACs, indications, and duration of follow up. Finally, 12 RCTs involving 89,549 patients were included, among which 44,816 (50%) receiving NOACs and 44,733 (50%) receiving warfarin. Overall, 1,139 (1.3%) patients including 515 NOACs users (1.1%) and 624 warfarin users (1.4%) developed fracture. Risk of fracture was significantly lower in NOACs compared to warfarin (RR: 0.82, 95%CI: 0.73–0.93, *P* = 0.001), with a NNT of 333. No significantly decreased risk was detected according to fracture sites. Subgroup analysis confirmed that the estimate of decreased fracture risk was derived mainly from AF patients receiving long-term anticoagulation treatment. The meta-regression did not detect any potential confounding on fracture risk. No heterogeneity between the studies (*I*^2^ = 15.0%) and no publication bias was identified. In conclusion, the use of NOACs was associated with a lower risk of fracture compared to warfarin, but with a relatively low absolute risk reduction. Therefore, screening for the fracture risk should be considered before initiating anticoagulation treatment. For patients who are at high risk of fracture or expected long-term treatment of anticoagulation, NOACs may represent a preferable alternative to warfarin.

## Introduction

Fracture is becoming more frequent than before with the aging of the world's population (Cummings and Melton, 2002). Risk factors, such as women, low bone density (LBD) and previous fracture, are well-known risk factors for fracture. Drugs including glucocorticoids, thyroid hormones, serotonin reuptake inhibitors, proton pump inhibitors, and vitamin K antagonists, were recognized to be associated with an increased fracture risk (Mazziotti et al., 2010). Warfarin, a vitamin K antagonist, that modulates the gamma-carboxylation of glutamic acid residues, was associated with LBD and it consequently led to an increased fracture risk (Sugiyama et al., 2015). Several studies have reported the potential link between warfarin use and increased fracture risk (Caraballo et al., 1999; Gage et al., 2006; Rejnmark et al., 2007).

Non-vitamin K Antagonist Oral Anticoagulants (NOACs)—dabigatran, apixaban, rivaoxaban, and edoxaban—that are either thrombin inhibitors or Xa factor inhibitors have been demonstrated to be non-inferior or superior to warfarin in terms of thromboprophylaxis and bleeding risk in phase III RCTs. Owing to their favorable net clinical benefit, international updated clinical guidelines have now issued a class I recommendation for the use of NOACs for stroke prevention in non-valvular atrial fibrillation (NVAF) patients (Kirchhof et al., 2016). In 2013, NOACs accounted for 62% of new anticoagulation prescriptions in United States among all cardiovascular prescriptions (Desai et al., 2014).

Interestingly, dabigatran had a better safety profile of bone than warfarin by increasing trabecular size and mineralization in rats (Fusaro et al., 2015). Rivaroxaban was proved not downgrade the fracture healing in a rat femur fracture model (Klüter et al., 2015). Moreover, edoxaban has no effects on the production of Gla-osteocalcin in a rat model (Morishima et al., 2013). These results implied that NOACs might have a lower risk of adverse effects on bone health than warfarin. Since the widespread use of NOACs in elderly people, fracture risk is becoming a key clinical issue. To our knowledge, only one study to date has stated the fracture risk with NOACs (dabigatran) vs. warfarin (Lau et al., 2017). In this population-based study, osteoporotic fracture developed in 104 (1.3%) patients during follow-up, and the use of dabigatran was associated with a significantly lower risk of osteoporotic fracture when compared to warfarin (0.7 vs. 1.1 per 100 person-years; absolute risk difference per 100 person-years, −0.68 [95% CI, −0.38 to −0.86]; incidence rate ratio, 0.38 [95% CI, 0.22 to 0.66]). Accordingly, the present study aim to determine and compare the fracture risk in patients treated with NOACs or warfarin by pooling included randomized controlled trials (RCTs) data.

## Methods

### Data sources and searches

This study was reported in consist with standards that were outlined in the Cochrane Handbook and the PRISMA Statement for Systemic Reviews (Hutton et al., 2015; Wei et al., 2016). Medline, Embase, and Cochrane Library electronic databases were searched to identify all potential eligible trials from inception to Sep 30th, 2017 without language restriction. The following terms were used for searching: “dabigatran” or “Pradaxa” or “rivaroxaban” or “Xarelto” or “apixaban” or “Eliquis” or “edoxaban” or “Savaysa” or “Non-vitamin K Antagonist Oral Anticoagulants” or “novel oral anticoagulants” or “new oral anticoagulants” or “factor Xa inhibitors” or “factor IIa inhibitors” in combination with “clinical trial” or “controlled clinical trial” or “randomized controlled trials.” In addition, unpublished trials were identified from the ClinicalTrials.gov Website. The bibliographies of published studies were also scrutinized to ensure that all relevant trials were identified. Two reviewers (Z.G. and L.Z.) independently searched the databases, and all disagreements were resolved by consulting a third author (X.L.).

### Study selection and outcomes

Studies were included if they met the following criteria: (1) Only RCTs were included; (2) treatment had to involve NOACs and warfarin, and reported the fracture events. The primacy outcome was a composite of any fracture, combining any fracture events reported in the trial. The secondary outcomes included (1) fragility fracture by merging data of vertebral, hip, rib, and wrist fracture; (2) vertebral fracture by merging data of spinal compression fracture, lumbar vertebral fracture, thoracic vertebral fracture, cervical vertebral fracture, spinal fracture, fractured coccyx, and fractured sacrum; (3) all fracture sites. Two reviewers (Z.G. and L.Z.) independently evaluated all study titles and abstracts for determining eligibility. Thereafter, full text was retrieved and assessed the relevant possibility according to the inclusion. All discrepancies were resolved by consulting a third author (X.L.).

### Data extraction, quality evaluation, and bias assessment

Information were extracted using a pre-specified form, including trial name, publication year, condition, sample size, mean age, sex, creatinine clearance, intervention groups, study duration, and reported fracture sites. Detailed fracture data that was not reported in the publications was further extracted from the ClinicalTrials.gov website. It included acetabulum fracture, ankle fracture, avulsion fracture, cervical vertebral fracture, clavicle fracture, compression fracture, facial bones fracture, femoral neck fracture, femur fracture, fibula fracture, foot fracture, forearm fracture, fracture, fractured coccyx, fractured sacrum, hand fracture, hip fracture, humerus fracture, jaw fracture, lower limb fracture, lumbar vertebral fracture, multiple fractures, open fracture, patella fracture, pelvic fracture, pubis Fracture, radius fracture, rib fracture, scapula fracture, skull fracture, spinal compression fracture, spinal fracture, sternal fracture, thoracic vertebral fracture, tibia fracture, upper limb fracture, and wrist fracture. Traumatic fracture was excluded from the analyses. The methodological quality of included RCTs was evaluated using Cochrane Collaboration Risk of Bias Tool (Higgins et al., 2011). Potential publication bias was evaluated by visually inspecting funnel plots (Wei et al., 2016).

### Data analysis

Relative ratios (RRs) and their 95% confidence intervals (CIs), according to fracture site, were calculated using a random- or fixed-effects model on the basis of between-study heterogeneity. Heterogeneity, defined as variation beyond chance, was assessed through *I*^2^ test and Q statistic. *I*^2^ of >50% indicated considerable heterogeneity, and a *p*-value of <0.05 at Q statistic represented a significant heterogeneity (Higgins et al., 2003). A fixed-effects model was used based on Mantel-Haenszel method unless heterogeneity was present. The number of patients needed to treat (NNT) to prevent 1 event was calculated as: (1/absolute risk reduction)×100, where absolute risk reduction was rate difference (event rates on warfarin minus event rates on NOACs). Subgroup analyses were conducted according to individual NOACs (dabigatran, rivaroxaban, apixaban, and edoxaban), indications (atrial fibrillation or venous thromboembolism), and duration of follow up (>1 year or <1 year). To explore the potential effect modifiers on outcomes, meta-regression analysis was performed to test demographic characteristics of the included RCTs. Sensitivity analyses were performed to identify the effect of a single trial by sequential elimination of each trial from the pool. In addition, further analyses were conducted to identify the effect by including the low-dose arms (dabigatran 110 mg in RE-LY, and edoxaban 30/15 mg in ENGAGE AF-TIMI 48). All statistical analyses were performed by using STATA software (version13, Statacorp, College Station, Texas, USA), and *P* < 0.05 indicated a statistically significant difference.

## Results

### Study evaluation

**Figure 1:**
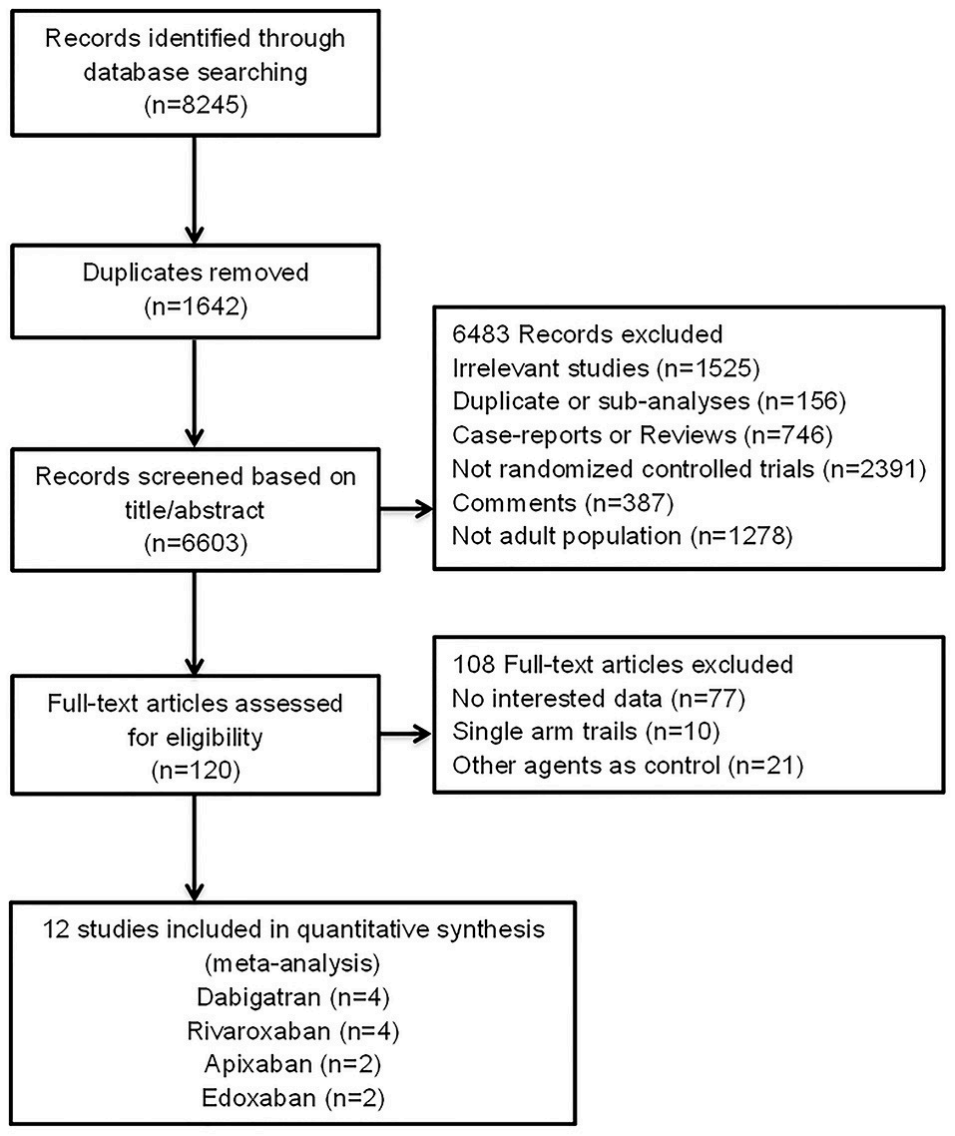
Flow diagram for the selection of eligible randomized controlled trials.

In total, 8,245 records were identified from the initial database search. After the removal of 1,642 duplicates, 6,483 records were excluded for various reasons through title and abstract screening. The remaining 120 records were full-text articles, of which 108 proved ineligible due to the unavailability of fracture data, single arm studies, or not warfarin as comparison. Finally, 12 eligible RCTs were included in the analyses (Figure 1 and Table S1) (Connolly et al., 2009; Schulman et al., 2009, 2013, 2014; EINSTEIN Investigators et al., 2010; Granger et al., 2011; Patel et al., 2011; EINSTEIN–PE Investigators et al., 2012; Hori et al., 2012; Agnelli et al., 2013; Giugliano et al., 2013; Hokusai-VTE Investigators et al., 2013). The characteristics of included RCTs were summarized in Tables 1, 2. Publication year varied from 2009 to 2014, with trial duration ranging from 3 to 36 months. A total of 89,549 patients were enrolled, among which 44,816 (50%) patients were treated with NOACs and 44,733 (50%) patients were treated with warfarin. Of these 12 trials, 5 (59,735 patients) were Atrial fibrillation (AF) studies, and 7 (29,814 patients) were venous thromboembolism (VTE) studies. All trials satisfied bias tool items with the exception of RE-LY (Connolly et al., 2009), EINSTEIN (EINSTEIN Investigators et al., 2010), and EINSTEIN-PE (EINSTEIN–PE Investigators et al., 2012), which were not double-blinded (Table S2).

**Table 1:** Summarized Characteristics.

**Source**	**Condition /Follow up (months)**	**Interventions**	**Number (*n*)**	**Age (year)/Male (%)**	**CrCL (ml/min)/CrCL30-50 ml/min (%)**	**Reported fracture site**
RE-LY, 2009	AF/24	Dabigatran 150 mg twice daily	6,059	71.5/63.2	NA/20.4	Ankle fracture; Cervical vertebral fracture; Clavicle fracture; Compression fracture; Facial bones fracture; Femoral neck fracture; Femur fracture; Fibula fracture; Foot fracture; Forearm fracture; Fracture; Hand fracture; Hip fracture; Humerus fracture; Lower limb fracture; Lumbar vertebral fracture; Multiple fractures; Patella fracture; Pelvic fracture; Pubis Fracture; Radius fracture; Rib fracture; Scapula fracture; Skull fracture; Spinal compression fracture; Spinal fracture; Sternal fracture; Thoracic vertebral fracture; Tibia fracture; Upper limb fracture; Wrist fracture
		Dabigatran 110 mg twice daily	5,983	71.4/64.3	NA/20.1	
		Warfarin	5,998	71.6/63.3	NA/18.9	
ROCKET AF, 2011	AF/23	Rivaroxaban 20 mg daily	7,111	73.0/60.3	NA/ 21.0	Ankle fracture; Avulsion fracture; Cervical vertebral fracture; Clavicle fracture; Compression fracture; Facial bones fracture, Femoral neck fracture; Femur fracture; Fibula fracture; Foot fracture; Fracture; Hand fracture; Hip fracture; Humerus fracture; Lower limb fracture; Lumbar vertebral fracture; Multiple fractures; Patella fracture; Pelvic fracture; Pubis Fracture; Radius fracture; Rib fracture; Spinal compression fracture; Spinal fracture; Thoracic vertebral fracture; Tibia fracture; Upper limb fracture; Wrist fracture
		Warfarin	7,125	73.0/60.3	NA/ 20.6	
J-ROCKET, 2012	AF/30	Rivaroxaban 15 mg daily	639	71.0/82.9	NA/22.1	Femur fracture; Fibula fracture; Patella fracture; Radius fracture; Rib fracture; Skull fracture; Spinal compression fracture; Tibia fracture; Ulna fracture
		Warfarin	639	71.2/78.2	NA/22.4	
ARISTOTLE, 2011	AF/21	Apixaban 5 mg twice daily	9,088	70.0/64.5	NA/15.0	Acetabulum fracture; Ankle fracture; Cervical vertebral fracture; Clavicle fracture; Facial bones fracture; Femoral neck fracture; Femur fracture; Fibula fracture; Foot fracture; Forearm fracture; Fracture; Hand fracture; Hip fracture; Lower limb fracture; Lumbar vertebral fracture; Open fracture; Patella fracture; Pelvic fracture; Pubis Fracture; Radius fracture; Rib fracture; Scapula fracture; Skull fracture; Spinal compression fracture; Spinal fracture; Sternal fracture; Thoracic vertebral fracture; Tibia fracture; Ulna fracture; Upper limb fracture; Wrist fracture
		Warfarin	9,052	70.0/65.0	NA/15.2	
ENGAGE AF-TIMI 48, 2013	AF/33	Edoxaban 60 mg daily	7,012	72.0/62.1	NA/19.6	Acetabulum fracture; Ankle fracture; Cervical vertebral fracture; Clavicle fracture; Compression fracture; Facial bones fracture; Femoral neck fracture; Femur fracture; Fibula fracture; Foot fracture; Forearm fracture; Fracture; Fractured coccyx; Hand fracture; Hip fracture; Humerus fracture; Jaw fracture; Lower limb fracture; Lumbar vertebral fracture; Multiple fractures; Patella fracture; Pelvic fracture; Pubis Fracture; Radius fracture; Rib fracture; Scapula fracture; Skull fracture; Spinal compression fracture; Spinal fracture; Sternal fracture; Thoracic vertebral fracture; Tibia fracture; Ulna fracture; Upper limb fracture; Wrist fracture
		Edoxaban 30 mg daily	7,002	72.0/61.2	NA/19.0	
		Warfarin	7,012	72.0/62.5	NA/19.3	
RE-COVER, 2009	VTE/6	Dabigatran 150 mg twice daily	1,273	56.0/58.0	105.8/NA	Femur fracture; Hip fracture; Lower limb fracture; Radius fracture; Rib fracture; Tibia fracture
		Warfarin	1,266	55.0/58.9	104.4/NA	
RE-COVER II, 2014	VTE/6	Dabigatran 150 mg twice daily	1,280	54.7/61.0	108.2/NA	Femoral neck fracture; Hip fracture; Humerus fracture; Multiple fractures; Upper limb fracture
		Warfarin	1288	55.1/60.2	107.1/NA	
RE-MEDY, 2013	VTE/6-36	Dabigatran 150 mg twice daily	1,430	55.4/60.9	104.2/NA	Acetabulum fracture; Ankle fracture; Femoral neck fracture; Femur fracture; Fibula fracture; Foot fracture; Hand fracture; Hip fracture; Humerus fracture; Lower limb fracture; Radius fracture; Tibia fracture; Upper limb fracture
		Warfarin	1,426	53.9/61.1	106.6/NA	
EINSTEIN, 2010	VTE/3, 6, or 12	Rivaroxaban 15 mg twice daily for 3 weeks, followed by 20 mg daily	1,718	55.8/57.4	NA/6.6	Ankle fracture; Clavicle fracture; Femoral neck fracture; Femur fracture; Humerus fracture; Radius fracture; Rib fracture; Spinal compression fracture; Thoracic vertebral fracture; Ulna fracture
		Warfarin	1,711	56.4/56.3	NA/7.0	
EINSTEIN-PE, 2012	PE/3, 6, or 12	Rivaroxaban 15 mg twice daily for 3 weeks, followed by 20 mg daily	2,412	57.9/54.1	NA/8.6	Ankle fracture; Cervical vertebral fracture; Facial bones fracture; Femoral neck fracture; Femur fracture; Fibula fracture; Foot fracture; Hip fracture; Humerus fracture; Lumbar vertebral fracture; Rib fracture; Spinal compression fracture; Sternal fracture; Thoracic vertebral fracture; Tibia fracture; Upper limb fracture
		Warfarin	2,405	57.5/51.7	NA/7.9	
AMPLIFY, 2013	VTE/6	Apixaban 10 mg twice daily for 7 days, followed by 5 mg twice daily	2,676	57.2/58.3	NA/6.0	Ankle fracture; Cervical vertebral fracture; Facial bones fracture; Femur fracture; Hip fracture; Humerus fracture; Lower limb fracture; Lumbar vertebral fracture; Pelvic fracture; Radius fracture; Spinal compression fracture; Upper limb fracture; Wrist fracture
		Warfarin	2,689	56.7/59.1	NA/5.5	
Hokusai-VTE, 2013	VTE/12	Edoxaban 60 mg daily	4,118	55.7/57.3	NA/6.5	Acetabulum fracture; Ankle fracture; Clavicle fracture; Compression fracture; Facial bones fracture; Femoral neck fracture; Femur fracture; Fibula fracture; Foot fracture; Forearm fracture; Fractured sacrum; Hand fracture; Hip fracture; Humerus fracture; Jaw fracture; Lower limb fracture; Lumbar vertebral fracture; Multiple fractures; Pelvic fracture; Pubis Fracture; Radius fracture; Rib fracture; Spinal compression fracture; Spinal fracture; Thoracic vertebral fracture; Tibia fracture; Upper limb fracture; Wrist fracture
		Warfarin	4,122	55.9/57.2	NA/6.6	

*AF, atrial fibrillation; CrCL, creatinine clearance; NA, not available; PE, pulmonary embolism; VTE, venous thromboembolism; RE-LY, Randomized Evaluation of Long-term Anticoagulation Therapy; ROCKET AF, Rivaroxaban Once Daily Oral Direct Factor Xa Inhibition Compared with Vitamin K Antagonism for Prevention of Stroke and Embolism Trial in Atrial Fibrillation; ARISTOTLE, Apixaban for Reduction in Stroke and Other Thromboembolic Events in Atrial Fibrillation; ENGAGE AF, Effective Anticoagulation with Factor Xa Next Generation in Atrial Fibrillation; EINSTEIN, Oral Direct Factor Xa Inhibition Rivaroxaban in Patients With Acute Symptomatic Deep Vein Thrombosis; EINSTEIN-PE, Oral Direct Factor Xa Inhibition Rivaroxaban in Patients With Acute Symptomatic Pulmonary Embolism*.

**Table 2:** Patient demographics and clinical characteristics of included studies.

**Source**	**Total number**	**Mean age (year)**	**Male (%)**	**Mean weight (kg)**	**Weight >100 kg (%)**	**Stroke/TIA**	**HF**	**Diabetes**	**Hypertension**	**CrCL (ml/min)**	**CrCL30-50 ml/min (%)**	**Cancer (%)**
RE-LY, 2009	18,040	71.5	63.6	82.7	NA	20.0	32.0	23.3	78.9	NA	19.8	NA
ROCKET AF, 2011	14,236	73.0	60.3	NA	NA	54.7	62.4	39.9	90.6	NA	20.8	NA
J-ROCKET, 2012	1,278	71.1	80.6	NA	NA	63.6	40.8	38.1	79.5	NA	22.3	NA
ARISTOTLE, 2011	18,140	70.0	64.7	82.0	NA	19.4	35.5	25.0	87.4	NA	15.1	NA
ENGAGE AF-TIMI 48, 2013	21,026	72.0	38.1	28.3	NA	NA	57.4	36.1	93.6	NA	19.3	NA
RE-COVER, 2009	2,539	54.7	58.4	84.9	NA	NA	NA	NA	NA	105.1	NA	4.8
RE-COVER II, 2014	2,568	54.9	60.6	83.0	NA	NA	NA	NA	NA	107.6	NA	3.9
RE-MEDY, 2013	2,856	54.7	61.0	86.1	NA	NA	NA	9.1	38.6	105.4	NA	4.2
EINSTEIN, 2010	3,429	56.1	56.9	NA	14.2	NA	NA	NA	NA	NA	6.8	6.0
EINSTEIN-PE, 2012	4,817	57.7	52.9	NA	14.6	NA	NA	NA	NA	NA	8.3	4.6
AMPLIFY, 2013	5,365	56.9	58.7	84.6	19.3	NA	NA	NA	NA	NA	5.7	2.7
Hokusai-VTE, 2013	8,240	55.8	57.2	NA	15.4	NA	NA	NA	NA	NA	6.6	9.4

*AF, atrial fibrillation; CrCL, creatinine clearance; NA, not available; PE, pulmonary embolism; VTE, venous thromboembolism; HF, heart failure; TIA, transient ischemic attack; RE-LY, Randomized Evaluation of Long-term Anticoagulation Therapy; ROCKET AF, Rivaroxaban Once Daily Oral Direct Factor Xa Inhibition Compared with Vitamin K Antagonism for Prevention of Stroke and Embolism Trial in Atrial Fibrillation; ARISTOTLE, Apixaban for Reduction in Stroke and Other Thromboembolic Events in Atrial Fibrillation; ENGAGE AF, Effective Anticoagulation with Factor Xa Next Generation in Atrial Fibrillation; EINSTEIN, Oral Direct Factor Xa Inhibition Rivaroxaban in Patients With Acute Symptomatic Deep Vein Thrombosis; EINSTEIN-PE, Oral Direct Factor Xa Inhibition Rivaroxaban in Patients With Acute Symptomatic Pulmonary Embolism*.

### Risk of any fracture

**Figure 2:**
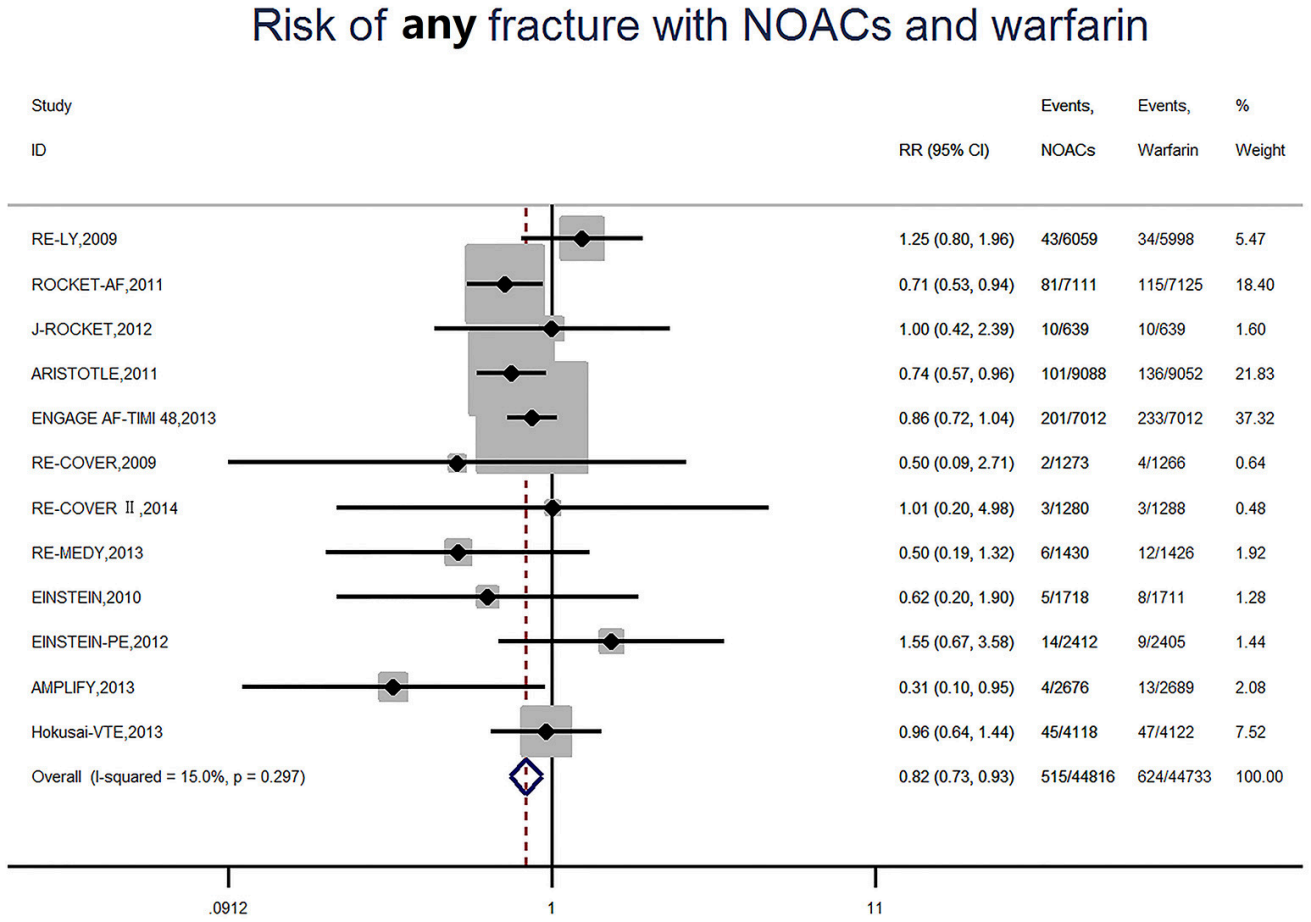
Risk of any fracture with Non-vitamin K antagonist oral anticoagulants and warfarin. RR indicates relative risk; 95%CI indicates 95% confidence interval.

A total of 1,139 (1.3%) developed fracture, of which 515 (1.1%) were NOACs users and 624 (1.4%) were warfarin users. Consequently, NOACs significantly reduced the risk of fracture by 18% (RR: 0.82, 95%CI: 0.73–0.93, *P* = 0.001) compared to warfarin (Figure 2), with no significant heterogeneity among included studies (*I*^2^ = 15.0%, *P* = 0.30). The data translated to NNT of 333, meaning that 333 patients treated with NOACs prevent 1 fracture event than those treated with warfarin. Among included studies, a high incidence of 2.9% (201 of 7,012) was found in the ENGAGE AF-TIMI 48 trial (AF trial and 33 months of follow up) (Giugliano et al., 2013), and the AMPLIFY study showed the low incidence of 0.1% (4 of 2676) in patients with NOACs (VET trial and 6 months of follow up) (Agnelli et al., 2013).

### Risk of fracture at different skeletal site

Risk of fracture at different site was presented in Table 3. With respect to fragility fracture, 212 (0.47%) occurred in patients receiving NOACs and 240 (0.54%) occurred in patients receiving warfarin. Therefore, the risk of fracture was numerically lower with NOACs, but this did not meet statistical significance (RR: 0.88, 95%CI: 0.73–1.06, *P* = 0.18). Regarding vertebral fracture, a reduced trend was found in patients using NOACs compared patients using warfarin (RR: 0.79, 95%CI: 0.59–1.06, *P* = 0.11). As to hip fracture, no significant difference was detected between NOACs-treated patients and warfarin-treated patients due to low incidence (RR: 0.99, 95%CI: 0.72–1.34, *P* = 0.93). Similar result was found among other fracture site.

**Table 3:** Relative risk of fracture at different skeletal site.

**Fracture site**	**No. of studies**	**With NOACs therapy**	**With warfarin therapy**	**RR**	**95%CI (*p*-value)**
Fragility fracture ^#^	12	212/44816(0.47%)	240/44733(0.54%)	0.88	0.73–1.06(0.18)
Vertebral fracture^##^	9	78/40833(0.19%)	99/40753(0.24%)	0.79	0.59–1.06(0.11)
Hip fracture	10	78/42459(0.18%)	79/42383(0.19%)	0.99	0.72–1.34(0.93)
Femur fracture	11	64/43536(0.15%)	79/43445(0.18%)	0.82	0.59–1.13(0.22)
Rib fracture	9	44/39430(0.11%)	51/39330(0.13%)	0.87	0.58–1.29(0.48)
Femoral neck fracture	9	44/40228(0.11%)	44/40139(0.11%)	1.00	0.66–1.51(1.00)
Spinal compression fracture	9	39/40883(0.10%)	42/40753(0.10%)	0.93	0.60–1.43(0.74)
Humerus fracture	9	31/33816(0.09%)	24/33776(0.07%)	1.27	0.76–2.12(0.36)
Ankle fracture	9	29/41624(0.07%)	37/41540(0.09%)	0.79	0.49–1.27(0.33)
Upper limb fracture	9	19/41186(0.05%)	28/41117(0.07%)	0.7	0.40–1.22(0.21)
Lumbar vertebral fracture	7	13/38476(0.03%)	20/38403(0.05%)	0.69	0.36–1.31(0.26)
Lower limb fracture	8	13/38767(0.03%)	18/38690(0.05%)	0.75	0.38–1.46(0.40)
Pelvic fracture	6	14/36064(0.04%)	15/35998(0.04%)	0.93	0.46–1.91(0.85)
Facial bones fracture	7	13/38476(0.03%)	15/38403(0.04%)	0.88	0.43–1.78(0.72)
Tibia fracture	9	15/39142(0.04%)	16/39045(0.04%)	0.95	0.50–1.80(0.87)
Foot fracture	7	14/37230(0.04%)	11/37140(0.03%)	1.23	0.59–2.55(0.58)
Pubis Fracture	5	11/33388(0.03%)	12/33309(0.04%)	0.92	0.41–2.05(0.84)
Thoracic vertebral fracture	7	7/37518(0.02%)	16/37425(0.04%)	0.50	0.22–1.11(0.09)
Wrist fracture	6	12/36064(0.03%)	11/35998(0.03%)	1.08	0.49–2.37(0.84)
Radius fracture	10	10/41124(0.02%)	13/41040(0.03%)	0.82	0.40–1.68(0.58)
Cervical vertebral fracture	6	6/34358(0.02%)	13/34281(0.04%)	0.52	0.21–1.25(0.14)
Spinal fracture	5	11/33388(0.03%)	8/33309(0.02%)	1.35	0.56–3.28(0.51)
Fibula fracture	8	6/37869(0.02%)	7/37779(0.02%)	0.89	0.35–2.25(0.81)
Clavicle fracture	6	3/35106(0.01%)	10/35020(0.03%)	0.42	0.15–1.18(0.10)
Hand fracture	6	4/34818(0.01%)	9/34735(0.03%)	0.52	0.19–1.47(0.22)
Patella fracture	5	6/29909(0.02%)	6/29826(0.02%)	1.00	0.36–2.75(1.0)
Forearm fracture	4	3/26277(0.01%)	6/26184(0.02%)	0.54	0.15–1.97(0.35)
Multiple fractures	5	3/25580(0.01%)	7/25545(0.03%)	0.56	0.19–1.66(0.29)
Ulna fracture	4	3/18457(0.02%)	4/18414(0.02%)	0.82	0.23–2.84(0.75)
Acetabulum fracture	4	4/21648(0.02%)	3/21612(0.01%)	1.22	0.35–4.24(0.75)
Skull fracture	4	1/22798(0.00%)	6/22701(0.03%)	0.29	0.06–1.37(0.12)
Sternal fracture	4	1/24571(0.00%)	7/24467(0.03%)	0.25	0.05–1.18(0.08)
Compression fracture	4	3/24300(0.01%)	3/24257(0.01%)	1.00	0.25–4.00(1.00)
Fracture	4	4/29270(0.01%)	1/29187(0.00%)	1.99	0.50–7.98(0.33)
Jaw fracture	2	4/11130(0.04%)	0/11134(0.00%)	5.00	0.58–42.81(0.14)
Scapula fracture	3	1/22159(0.00%)	2/22062(0.01%)	0.60	0.08–4.53(0.62)
Avulsion Fracture	1	1/7111(0.01%)	0/7125(0.00%)	3.01	0.12–73.77(0.50)
Fractured COCCYX	1	1/7012(0.01%)	0/7012(0.00%)	3.00	0.12–73.63(0.50)
Open fracture	1	0/9088(0.00%)	1/9052(0.01%)	0.33	0.01–8.15(0.50)
Fractured sacrum	1	1/4118(0.02%)	0/4122(0.00%)	3.00	0.12–73.69(0.50)

NOACs, Non-vitamin K Antagonist Oral Anticoagulants; RR, relative risk; 95%CI, 95% confidence interval;

# data of vertebral fracture, hip fracture, rib fracture, and wrist fracture was merged;

## *data of spinal compression fracture, lumbar vertebral fracture, thoracic vertebral fracture, cervical vertebral fracture, spinal fracture, fractured coccyx, and fractured sacrum was merged as vertebral fracture*.

### Risk of fracture based on subgroup

According to each NOACs, as shown in Table 4, rivaroxaban (RR: 0.78, 95%CI: 0.61–0.99, *P* = 0.04) and apixaban (RR: 0.70, 95%CI: 0.55–0.90, *P* = 0.01) showed a lower fracture risk when compared to warfarin, and no significant difference was observed for dabigatran and edoxaban (*P* for interaction among different NOACs: 0.33). In AF patients, a significantly lower fracture risk was detected in NOACs vs. warfarin (RR: 0.82, 95%CI: 0.73–0.93, *P* < 0.01), with a NNT of 333. No significant result was observed in patients with VTE (P for interaction between indication: 1.00). In patients who received long-term anticoagulation treatment (>1 year), NOACs significantly reduced the risk of fracture compared with warfarin (RR: 0.82, 95%CI: 0.73–0.93, *P* < 0.01). The similar results were not found in patients with short-term treatment (*P* for interaction between treatment duration: 0.74).

**Table 4:** Subgroup analyses.

**Subgroup**	**No. of studies**	**With NOACs therapy**	**With warfarin therapy**	**RR**	**95%CI (*p*-value)**	**Homogeneity**		**NNT**	***P* for interaction**
						***I*^2^ (%)**	***p*-value**		
**NOACs**									0.33
Dabigatran	4	54/10042(0.5%)	53/9978(0.5%)	1.01	0.69–1.48(0.96)	15.6	0.31	—	
Rivaroxaban	4	110/11880(0.9%)	142/11880(1.2%)	0.78	0.61–0.99(0.04)	15.5	0.31	333	
Apixaban	2	105/11764(0.9%)	149/11741(1.3%)	0.70	0.55–0.90(0.01)	55.0	0.14	250	
Edoxaban	2	246/11130(2.2%)	280/11134(2.5%)	0.88	0.74–1.04(0.13)	0.0	0.64	—	
**Indication**									1.00
Atrial fibrillation	5	436/29909(1.5%)	528/29826(1.8%)	0.82	0.73–0.93(<0.01)	28.7	0.23	333	
VTE	7	79/14907(0.5%)	96/14907(0.6%)	0.82	0.61–1.11(0.20)	18.2	0.29	—	
**Treatment duration**									0.74
>1 year	5	436/29909(1.5%)	528/29826(1.8%)	0.82	0.73–0.93(<0.01)	28.7	0.23	333	
<1 year	6	73/13477(0.5%)	84/13481(0.6%)	0.87	0.64–1.19(0.38)	18.5	0.29	—	

*NOACs, Non-vitamin K Antagonist Oral Anticoagulants; RR, relative risk; 95%CI, 95% confidence interval; VTE, venous thromboembolism NNT, number of patients needed to treat*.

### Sensitivity analyses and meta-regression

The overall outcomes failed to identify any individual trials as having influenced the results to a significant extent (Table S3). When low-dose regimen in RE-LY and ENGAGE AF-TIMI 48 trials was included in the analyses, the result was consistent with the primacy analysis (Figure S1). Similarly, the meta-regression analysis failed to identify any potential confounding on fracture risk (Table S4).

### Publication bias

Visual inspection of funnel plots for the analyses showed that all plots exhibited fairly symmetrical inverted funnel shapes, suggesting that publication bias was not a concern (Figure S2).

## Discussion

Previous studies have stated the potential link between warfarin use and increased fracture risk. At present, NOACs provide an improved clinical net benefit compared with warfarin, and are recommended by international clinical guideline. With the widespread use of NOACs in elderly people, fracture risk is becoming a key clinical issue. However, the risk of fracture in patients receiving NOACs or warfarin is unclear. For this reason, we have performed the first systematic review to pool current evidence for analyzing the risk of fracture in patients with NOACs therapy. The results indicated that the use of NOACs was associated with a lower fracture risk when compared to warfarin, but with a relatively low absolute risk reduction.

### Potential mechanism for fracture

Several reasons might explain the reason of relatively lower fracture risk in NOACs than warfarin. Firstly, recent studies have investigated the effects of NOACs on bone biology. An *in vivo* study indicated that dabigatran had an increased bone volume, decreased trabecular separation and lower bone turnover rate compared to that observed in warfarin-treated rats (Fusaro et al., 2015). In addition, rivaroxaban revealed a larger callus and a marginal increase of the bone tissue mineral density in a rat femur fracture model than control group, which demonstrated that rivaroxaban could conduct a positive effect on fracture healing (Klüter et al., 2015). Similarly, edoxaban had no effects on total, undercarboxylated-, and Gla-osteocalcin levels even at a high dosage of 54 mg/kg rats, while warfarin impaired the carboxylation of osteocalcin (Morishima et al., 2013). These results revealed that NOACs might have protective effect on bone health. In contrast, warfarin may interfere with the processes of bone formation (Sugiyama et al., 2015). It is a vitamin K antagonist and produces its anticoagulant effect by modulating carboxylation reaction in vitamin K-dependent clotting proteins (Sugiyama et al., 2015). In fact, warfarin also disturbs the carboxylation of vitamin K-dependent bone proteins, including osteocalcin, matrix Gla protein, and periostin (Tufano et al., 2015), which play an important role in bone mineralization (Tufano et al., 2015). An increased undercarboxylated osteocalcin levels as well as fracture risk in warfarin-treated patients were demonstrated in previous studies (Gage et al., 2006). Accordingly, NOACs might be associated with a lesser effect on bone health compared to warfarin because of the little effect on vitamin K.

Secondly, in order to achieve a satisfied anticoagulation effect, dietary restrictions of vitamin K rich foods was frequently adopted in patients receiving warfarin. In our included RCTs, good control of anticoagulation (as reflected by average time in therapeutic range, TTR) may be related to dietary limitation. Vitamin K1, mainly provided by leafy and green vegetables, is involved in multiple stages on bone metabolism and poor vitamin status is linked to low bone mass and high fracture risk (Pearson, 2007). NOACs users, without dietary restrictions, are less likely to develop fracture events related to vitamin K deficiency.

Although several animal studies have provided the positive effects of NOACs on bone biology. No similar studies have been conducted in humans. In addition, vitamin K1 concentrations were not detected in warfarin-treated patients who had been strictly limited in consumption of Vitamin K rich foods. Thus, the exact mechanism of NOACs on human bones is still necessary to be explored.

### In comparison to other studies

Up to now, only one study has described the fracture risk with NOACs vs. warfarin (Lau et al., 2017). In this population-based retrospective cohort study, propensity score matching was used to exclude patients with a high tendency of taking dabigatran or warfarin from the comparison. Therefore, the results were less likely to lead bias due to residual confounding. It is in line with our finding that patients treated with NOACs had a lower risk of fracture compared with patients treated with warfarin. Notably, absolute risk reduction is correspondingly low between NOACs and warfarin, with NNT of 333. It means that 333 patients taking NOACs only prevent 1 fracture event than those taking warfarin. Regarding the relationship between warfarin use and fracture risk, as summarized in Table S5, previous studies have had conflicting results. After intensive analysis of each study, we draw a founding that those negative studies involved several limitations. Firstly, warfarin's adverse effects on fractures are relatively small and are presumed to be cumulative, so that small samples and/or short treatment duration might lead to an underestimation of fracture risk in warfarin users (Jamal et al., 1998; Sato et al., 2010; Misra et al., 2014). The similar result was also found in our subgroup analysis based on duration of follow up (<1 year or >1 year). Secondly, some studies only described the risk of fracture at specific sites (hip, spine, and wrist), which may lead to negatively statistical results due to low incidence (Mamdani et al., 2003; Sato et al., 2010; Misra et al., 2014). Furthermore, vertebral compression fracture is often asymptomatic and may not be diagnosed. Therefore, above-mentioned studies could not get a more complete picture in bone effects on warfarin. Thirdly, none of the studies collected relevant information on warfarin treatment, such as the adherence to warfarin and the quality of anticoagulation control (TTR) (Jamal et al., 1998; Mamdani et al., 2003; Pilon et al., 2004; Woo et al., 2008; Sato et al., 2010; Misra et al., 2014). The lack of such information might impede the comparability between warfarin users and non-users. Fourthly, as inherent in observational studies, concerns about residual confounding (history of falls, previous fracture, tobacco, alcohol, proton pump inhibitors, etc.,) remain even if those studies have adjusted the measured confounding factors. Therefore, the results might bias toward either direction. Finally, the underlying characteristics between warfarin users and non-users were likely to be different when referring to stroke risk and stroke itself is associated with an increased risk of fracture (Jamal et al., 1998; Woo et al., 2008; Sato et al., 2010; Misra et al., 2014; Benzinger et al., 2015). In the present study, as inherent in RCTs, interventions were comparable because of the same indication (AF or VTE), similar population characteristics, and strict follow up for warfarin. Importantly, in an attempt to obtain an authentic finding, we have pooled 12 large sample size RCTs and all the fracture events for a meaningful analysis. In addition, potential confounding on fracture risk was also tested using meta-regression analyses. Finally, our analyses showed a significantly lower fracture risk of NOACs compared with warfarin in spite of the presence of low absolute risk reduction. Actually, the positive result was derived mainly from 5 AF studies that involved larger sample size (59,735 patients), longer duration of follow up (>1 year), and higher fracture incidence than those in VTE studies, which implied a potentially cumulative risk on fracture in patients taking warfarin. Furthermore, it is predictable that fracture risk when regarding different site was not significantly different between NOACs and warfarin owing to low incidence. With respect to individual NOACs, rivaroxaban and apixaban seem to be safer than warfarin. However, no significant interaction was detected among different NOACs.

### Clinical implications

Given that many risk factors for fracture (old age, stroke, previous fracture, etc.,) are also risk factors for thromboembolism among patients taking anticoagulation. The use of warfarin is at even greater risk of fracture in this fragile population. Thus, screening for the risk of fracture should be considered before initiating anticoagulation treatment and NOACs may represent a preferable alternative to warfarin in patients who are at high risk of fracture or expected long-term anticoagulation treatment.

### Limitations

Several limitations need to be considered. Firstly, four studies of NOACs were excluded from the analysis owing to the unavailable fracture data, which might reduce the power of statistics. Secondly, fracture data is notoriously difficult to ascertain, differently collected method of fracture data across trials might introduce certain bias. Thirdly, we have not get access to patient-level data in relation to relevant clinical characteristics, making powerful subgroup analysis unavailable. Whereas, we have performed a meta-regression analysis to assess potential effect modifiers in baseline characteristics, and the results failed to identify any potential confounding on fracture risk. Undeniably, residual confounding effects between included studies cannot be excluded absolutely. Fourthly, the mean duration of follow up was insufficiently long, which may underestimate the fracture risk of warfarin. Finally, included RCTs were not especially designed to assess the fracture risk of NOACs. Therefore, further RCTs as well as long-term observational studies are necessary to be conducted.

## Conclusions

The use of NOACs conferred a relatively lower risk of fracture compared to warfarin, with a very low absolute risk reduction. Hence, NOACs may represent a preferable alternative to warfarin in patients who are at high risk of fracture.

## Author contributions

X-YL, H-WL, and JP are the guarantors of the entire manuscript. Z-CG and L-YZ contributed to the study conception and design; data acquisition, analysis, and interpretation; drafting of the manuscript; critical revision of the manuscript for important intellectual content; and final approval of the version to be published. LS and CZ contributed to the data acquisition, analysis, and interpretation.

### Conflict of interest statement

The authors declare that the research was conducted in the absence of any commercial or financial relationships that could be construed as a potential conflict of interest.
