# Supplementary material for: Non-vitamin K Antagonist Oral Anticoagulants vs. Warfarin at Risk of Fractures: A Systematic Review and Meta-Analysis of Randomized Controlled Trials
Source: Front Pharmacol. 2018 Apr 10;9:348. doi: 10.3389/fphar.2018.00348 (PMC5903161; doi:10.3389/fphar.2018.00348)
Supplement: Supplementary file 1 [file Table1.DOCX]

Table S1. Reasons for excluded randomized controlled trials in ClinicalTrials.gov website

| NCT number | Phase | Study name | Drugs | Condition | Reason for exclusion |
| --- | --- | --- | --- | --- | --- |
| NCT01546883 | 4 | DEPAF | Dabigatran | AF | One arm study |
| NCT00808067 | 3 | RELY-ABLE | Dabigatran | AF | One arm study |
| NCT01184989 | 4 | NA | Dabigatran | Total knee or hip replacement | One arm study |
| NCT00558259 | 3 | RE-SONATE | Dabigatran | VTE | Placebo as control |
| NCT01896297 | 4 | NA | Dabigatran | AF and CKD | One arm study |
| NCT00657150 | 3 | NA | Dabigatran | Total hip arthroplasty | Enoxaparin as control |
| NCT00168818 | 3 | NA | Dabigatran | Hip replacement surgery | Enoxaparin as control |
| NCT00168805 | 3 | RE-MODEL | Dabigatran | Knee replacement surgery | Enoxaparin as control |
| NCT00152971 | 3 | NA | Dabigatran | Total knee replacement | Enoxaparin as control |
| NCT01493557 | 4 | NA | Dabigatran | AF | PPI as control |
| NCT01868243 | 2, 3 | DAWA | Dabigatran | AF with bioprosthesis replacement | Interested outcome unavailable |
| NCT01511939 | 3 | ATCCTCP-1 | Dabigatran | Topical pennsaid for knee pain | Interested outcome unavailable |
| NCT01094886 | 3 | NA | Rivaroxaban | Orthopedic surgery | One arm study |
| NCT01674647 | 3 | X-VERT | Rivaroxaban | AF and cardioversion | Interested outcome unavailable |
| NCT00439725 | 3 | Einstein-Extension | Rivaroxaban | VTE | Placebo as control |
| NCT00809965 | 3 | NA | Rivaroxaban | ACS | Placebo as control |
| NCT01729871 | 3 | VENTURE-AF | Rivaroxaban | AF with ablation | Interested outcome unavailable |
| NCT00362232 | 3 | RECORD 4 | Rivaroxaban | Total knee replacement | Enoxaparin as control |
| NCT00571649 | 3 | MAGELLAN | Rivaroxaban | Acute medical illness | Enoxaparin as control |
| NCT00457002 | 3 | ADOPT | Apixaban | Acute Medical Illness | Enoxaparin as control |
| NCT00496769 | 3 | AVERROES | Apixaban | AF | Aspirin as control |
| NCT00633893 | 3 | Amplify-ext | Apixaban | VTE | Placebo as control |
| NCT00452530 | 3 | ADVANCE-2 | Apixaban | Knee replacement surgery | Enoxaparin as control |
| NCT00423319 | 3 | ADVANCE-3 | Apixaban | Hip replacement surgery | Enoxaparin as control |
| NCT00371683 | 3 | NA | Apixaban | Knee replacement surgery | Enoxaparin as control |
| NCT01780987 | 3 | NA | Apixaban | VTE | Interested outcome unavailable |
| NCT00831441 | 3 | APPRAISE-2 | Apixaban | ACS | Placebo as control |
| NCT01706146 | 4 | REACT COM | Apixaban | AF | One arm study |
| NCT01857583 | 3 | NA | Edoxaban | Orthopedic surgery | Fondaparinux as control |
| NCT01181167 | 3 | NA | Edoxaban | Total hip arthroplasty | Enoxaparin as control |
| NCT01181102 | 3 | STARS E-3 | Edoxaban | Total knee arthroplasty | Enoxaparin as control |
| NCT01857622 | 3 | NA | Edoxaban | AF and CKD | One arm study |
| NCT01181141 | 3 | NA | Edoxaban | Hip fracture surgery | Enoxaparin as control |

ACS: acute coronary syndrome; AF: atrial fibrillation; CKD: chronic kidney disease; NA: not available; PPI: proton pump inhibitor; VTE: venous thromboembolism
