# Supplementary material for: Non-vitamin K Antagonist Oral Anticoagulants vs. Warfarin at Risk of Fractures: A Systematic Review and Meta-Analysis of Randomized Controlled Trials
Source: Front Pharmacol. 2018 Apr 10;9:348. doi: 10.3389/fphar.2018.00348 (PMC5903161; doi:10.3389/fphar.2018.00348)
Supplement: Supplementary file 2 [file Table2.DOCX]

Table S2. Quality assessment

| Study | Random sequence generation | Allocation concealment | Blinding of participants and personnel | Blinding of outcome assessment | Incomplete outcome data | Selective reporting | Other bias |
| --- | --- | --- | --- | --- | --- | --- | --- |
| RE-LY, 2009 | L | L | H | L | L | L | L |
| ROCKET AF, 2011 | L | L | L | L | L | U | L |
| J-ROCKET, 2012 | U | U | U | U | L | L | L |
| ARISTOTLE, 2011 | L | L | L | L | L | U | L |
| ENGAGE AF-TIMI 48, 2013 | L | L | L | L | L | L | L |
| RE-COVER, 2009 | L | L | L | L | L | L | L |
| RE-COVER Ⅱ, 2014 | L | U | L | L | L | L | L |
| RE-MEDY, 2013 | L | L | L | L | U | L | L |
| EINSTEIN, 2010 | L | U | H | L | L | L | L |
| EINSTEIN-PE, 2012 | L | U | H | L | L | L | L |
| AMPLIFY, 2013 | L | L | L | L | L | L | L |
| Hokusai-VTE, 2013 | L | U | L | L | L | L | L |

L: low risk; U: unclear risk; H: high risk
