# Supplementary material for: Non-vitamin K Antagonist Oral Anticoagulants vs. Warfarin at Risk of Fractures: A Systematic Review and Meta-Analysis of Randomized Controlled Trials
Source: Front Pharmacol. 2018 Apr 10;9:348. doi: 10.3389/fphar.2018.00348 (PMC5903161; doi:10.3389/fphar.2018.00348)
Supplement: Supplementary file 3 [file Table3.DOCX]

Table S3. Sensitivity analyses

| **Study omitted** | **RR** | **95%CI** |
| --- | --- | --- |
| RE-LY, 2009 | 0.80 | 0.71-0.90 |
| ROCKET AF, 2011 | 0.85 | 0.75-0.97 |
| J-ROCKET, 2012 | 0.82 | 0.73-0.92 |
| ARISTOTLE, 2011 | 0.85 | 0.74-0.97 |
| ENGAGE AF-TIMI 48, 2013 | 0.80 | 0.69-0.93 |
| RE-COVER, 2009 | 0.83 | 0.74-0.93 |
| RE-COVER Ⅱ, 2014 | 0.82 | 0.73-0.92 |
| RE-MEDY, 2013 | 0.83 | 0.74-0.93 |
| EINSTEIN, 2010 | 0.83 | 0.74-0.93 |
| EINSTEIN-PE, 2012 | 0.81 | 0.72-0.91 |
| AMPLIFY, 2013 | 0.84 | 0.74-0.94 |
| Hokusai-VTE, 2013 | 0.81 | 0.72-0.92 |

RR: relative risk; 95%CI: 95% confidence interval
