# Supplementary material for: Non-vitamin K Antagonist Oral Anticoagulants vs. Warfarin at Risk of Fractures: A Systematic Review and Meta-Analysis of Randomized Controlled Trials
Source: Front Pharmacol. 2018 Apr 10;9:348. doi: 10.3389/fphar.2018.00348 (PMC5903161; doi:10.3389/fphar.2018.00348)
Supplement: Supplementary file 4 [file Table4.DOCX]

Table S4. Meta-regression analyses

| Variable | P value |
| --- | --- |
| Age (year) | 0.201 |
| Male (%) | 0.410 |
| Weight (kg) | 0.451 |
| Weight>100kg (%) | 0.684 |
| Stroke/TIA | 0.884 |
| Heart failure | 0.724 |
| Diabetes | 0.645 |
| Hypertension | 0.364 |
| CrCL (ml/min) | 0.875 |
| CrCL30-50ml/min (%) | 0.472 |
| Cancer (%) | 0.329 |

P values: it is the results of meta-regression for the relationship between each variable and the outcomes; CrCL: creatinine clearance; TIA: transient ischemic attack
