# Supplementary material for: Non-vitamin K Antagonist Oral Anticoagulants vs. Warfarin at Risk of Fractures: A Systematic Review and Meta-Analysis of Randomized Controlled Trials
Source: Front Pharmacol. 2018 Apr 10;9:348. doi: 10.3389/fphar.2018.00348 (PMC5903161; doi:10.3389/fphar.2018.00348)
Supplement: Supplementary file 5 [file Table5.DOCX]

Table S5. The fracture risk of oral anticoagulants in published studies

| Source | Condition /Follow up (years) | Interventions/Number | Study type | Outcomes | Adjusted estimate (95%CI) | Adjusted covariates |
| --- | --- | --- | --- | --- | --- | --- |
| Jamal et al, 1998 | Elderly women/3.5 | Warfarin users/149; Nonusers/6052 | Prospective observational study | Fracture | HR:1.0 (0.6-1.7) | Self-reported health status, frailty on physical examination, involuntary weight loss, nonthiazide diuretics |
| Caraballo et al, 1999 | Women with VTE/NA | Oral anticoagulants/572 | Population-based retrospective cohort study | Vertebral fracture | SIR: 2.4 (1.6-3.4) | NA |
| Mamdani et al, 2003 | Elderly patients/NA | Warfarin users/52701 PPI; users/60383 | Population-based retrospective cohort study | Hip fracture | RR: 0.94 (0.81-1.09) | NA |
| Pilon et al, 2004 | Elderly patients/3.3 | Case/1523; control/15205 | Case-control study | Fracture | OR: 1.1 (0.9-1.6) | Sex, hyperthyroidism, corticosteroids, L-thyroxine, benzodiazepines, L-dopa, antidepressants, estrogens thiazide diuretics, statins |
| Gage et al, 2006 | Elderly patients with AF/>1 | Warfarin users/4461; Nonusers/7587 | Population-based retrospective cohort study | Hip, spine, or wrist fracture | OR: 1.25 (1.06-1.48) | Age, race, sex, beta-blocker, heart failure, high fall risk, hyperthyroidism, neuropsychiatric impairment, alcoholism, corticosteroid |
| Rejnmark et al, 2007 | NA/NA | Case/124655; control/373962 | Population-based case-control study | Fracture | OR: 1.1 (1.03-1.18) | Annual income, marital status, occupational status, charlson index, precious fracture, antiresorptive drug, corticosteroids, levothyroxine, antithyroid drugs, anxiolytics, sedatives, hypnotics, neuroleptics, antidepressants, antiepileptic drug, diuretics, statins, amiodarone |
| Woo et al,2008 | Elderly men  /5.1 | Warfarin users/321; Nonusers/5212 | Prospective cohort study | Nonspine fracture | HR: 1.06 (0.68-1.65) | Age, race, site, hypertension, cardiovascular disease, precious falls, self-reported health, diuretics, beta blockers |
| Sato et al, 2010 | Patients with previous hemispheric infarction and AF/5 | Warfarin users/70; Nonusers/83 | Prospective observational study | Hip fracture | P=0.92 | Age, sex, illness duration, severity of hemiplegia, barthel index |
| Misra et al, 2014 | Elderly patients with AF/>1 or >3 | Warfarin users/10173; Nonusers/10173 | Population-based retrospective cohort study | Hip, spine, and wrist fracture | >1 year, HR: 0.92 (03.77-1.1); >3 year, HR: 1.12 (0.88-1.42) | Age, sex, body mass index, high fall risk, DVT, PE, heart failure, neuropsychiatric impairment, hyperthyroidism, estrogen, beta blockers, corticosteroids, diuretics, bisphosphonates, statins, smoking, alcoholism |
| Lau et al, 2017 | AF/1.4 | Dabigatran/3298; Warfarin/6981 | Population-based retrospective cohort study | Hip and vertebral fracture | IRR: 0.38 (0.22-0.66) | Age, sex, index year, heart failure, ischemic stroke, transient ischemic attack, chronic obstructive pulmonary disease, diabetes mellitus, liver disease, osteoporosis, rheumatoid arthritis, chronic kidney disease, history of fall or fractures, angiotension-converting enzyme inhibitors or angiotensionⅡreceptor blockers, beta blockers, bisphosphonates, antidepressants, glucocorticoids |

AF: atrial fibrillation; DVT: deep venous thrombosis; HR: hazard ratio; IRR: incidence rate ratio; NA: not available; OR: odds ratio; PPI: proton pump inhibitor; PE: pulmonary embolism; RR: relative risk; SIR: standardized incidence ratio
