# Supplementary figures and images for: Non-vitamin K Antagonist Oral Anticoagulants vs. Warfarin at Risk of Fractures: A Systematic Review and Meta-Analysis of Randomized Controlled Trials
Source: Front Pharmacol. 2018 Apr 10;9:348. doi: 10.3389/fphar.2018.00348 (PMC5903161; doi:10.3389/fphar.2018.00348)

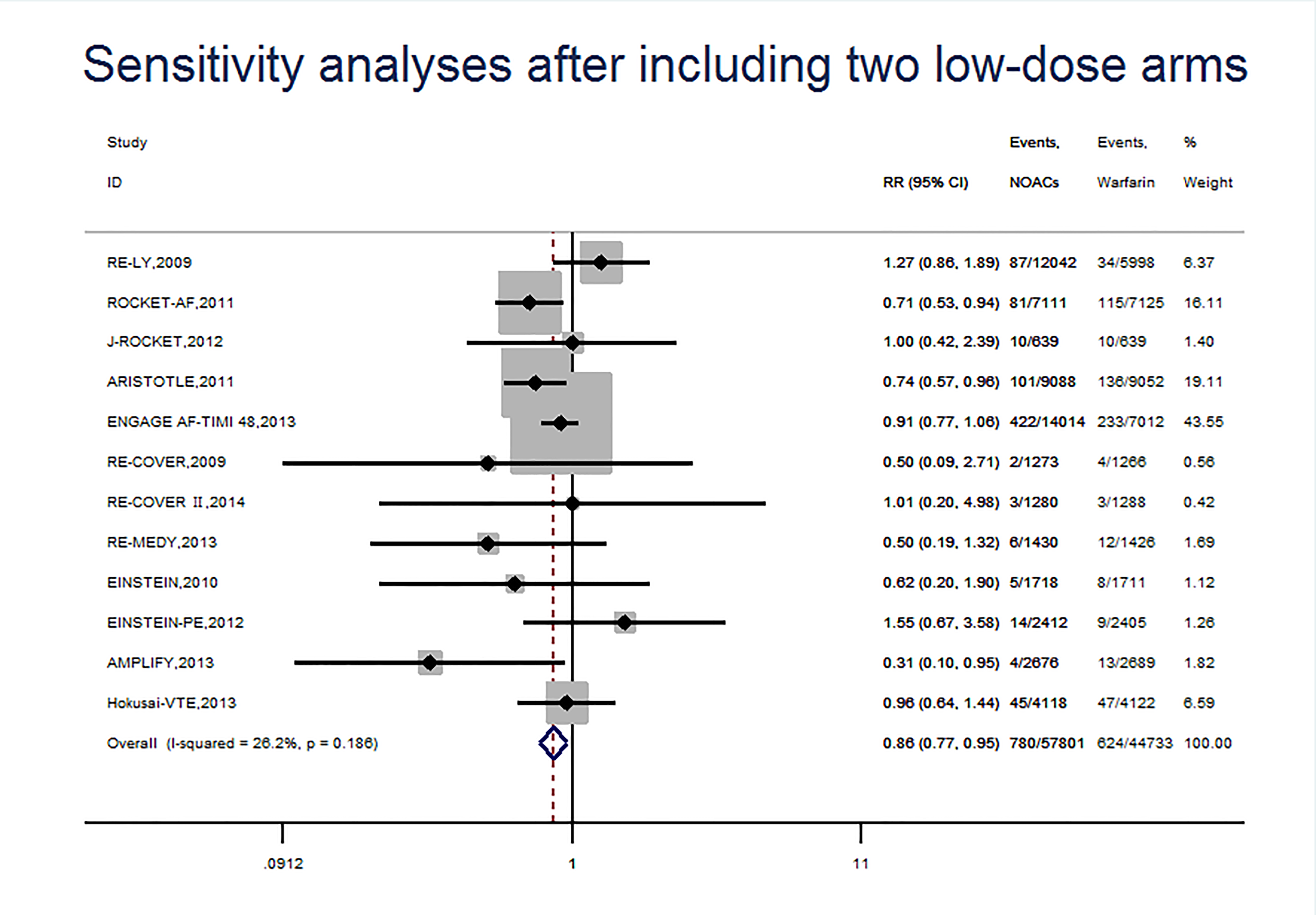

Supplement: Supplementary file 6 [file Image1.tif]

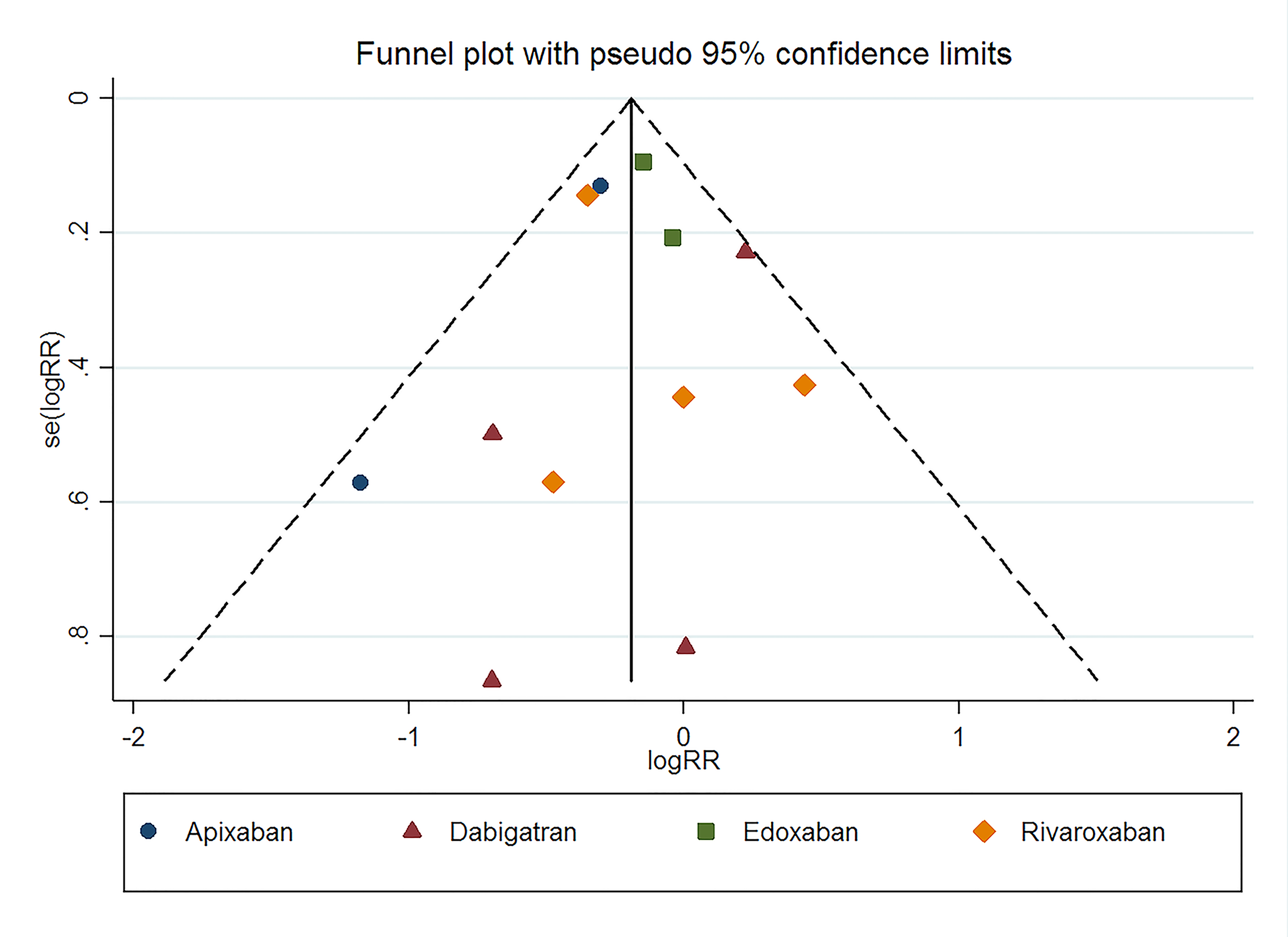

Supplement: Supplementary file 7 [file Image2.tif]
