# Supplementary material for: Non-vitamin K Antagonist Oral Anticoagulants vs. Warfarin at Risk of Fractures: A Systematic Review and Meta-Analysis of Randomized Controlled Trials
Source: Front Pharmacol. 2018 Apr 10;9:348. doi: 10.3389/fphar.2018.00348 (PMC5903161; doi:10.3389/fphar.2018.00348)
Supplement: Supplementary file 8 [file DataSheet1.docx]

**Figure Legends**

Figure S1: Sensitivity analyses after including two low-dose arms. RR indicates relative risk; 95%CI indicates confidence interval.

Figure S2. Funnel plot. logRR indicates the logarithmic value of relative risk; se(logRR) indicates the standard error of logRR.
